# The Influence of Companion Animals on Quality of Life of Gay and Bisexual Men Diagnosed with Prostate Cancer

**DOI:** 10.3390/ijerph16224457

**Published:** 2019-11-13

**Authors:** Morgan M. Wright, Pamela Schreiner, B. R. Simon Rosser, Elizabeth J. Polter, Darryl Mitteldorf, William West, Michael W. Ross

**Affiliations:** 1 Division of Epidemiology and Community Health, School of Public Health, University of Minnesota, Minneapolis, MN 55454, USA; schre012@umn.edu (P.S.); rosser@umn.edu (B.R.S.R.); polte004@umn.edu (E.J.P.); 2 Malecare Cancer Support, New York, NY 10001, USA; darrylm@malecare.org; 3 Department of Writing Studies, University of Minnesota, Minneapolis, MN 55454, USA; westx005@umn.edu; 4 Department of Family Medicine and community Health, Program of Human Sexuality, University of Minnesota, Minneapolis, MN 55454, USA; mwross@umn.edu

**Keywords:** health benefits, psychological benefits, pet companionship, cancer, prostate, sexual minorities

## Abstract

There has been almost no research on associations of companion animals with quality of life in sexual minorities. Because gay and bisexual men have less social support than their heterosexual peers, some have argued that pet companionship could provide emotional support, while others have argued the opposite, that having a pet is another stressor. This analysis examines the association between having dogs, cats, both animals, or no animals and quality of life using the 12-item Short Form (SF-12) mental and physical composite quality of life scores for gay and bisexual prostate cancer survivors, post-treatment. Participants were 189 gay, bisexual, or other men who have sex with men, who completed online surveys in 2015. Linear regression analysis found that participants with cats and participants with dogs had lower mental quality of life scores than participants without pets. After adjustment for covariates, mental health scores remained significantly lower for cat owners, dog owners, and owners of both animals compared to those of participants who did not have pets. No differences were seen for physical quality of life scores after adjustment. We conclude that pet companionship may be a net stressor for gay and bisexual men following prostate cancer treatment. As this is the first study of pet companionship in sexual minorities, further research is needed to confirm the reliability of these findings, generalizability, and temporality of the association.

## 1. Introduction

There is almost no research on the health effects of companion animals in sexual minority populations and none in gay and bisexual men with prostate cancer. Sixty-eight percent of the United States population has companion animals, colloquially known as pets [1]. The two most popular companion animals are dogs and cats. Of households with pets, 71% have dogs and 55% have cats [1]. Few organizations collect large-scale data about companion-ownership rates among sexual minorities. Community Marketing Inc. has conducted an annual lesbian, gay, bisexual, and transgender (LGBT) community marketing survey (*n* = 12,647) and found that 41% of gay men have dogs and 27% have cats [2]. 

There are several reasons why gay and bisexual men may turn to animals for companionship and support. Gay and bisexual men are more likely to be single and live alone and less likely to have children than heterosexual men [3,4]. In both qualitative interviews and quantitative surveys with gay and bisexual prostate cancer patients (conducted as part of this study), participants were more likely to undergo treatment alone than heterosexual prostate cancer patients were [5,6]. Thus, pets may play a larger role in providing companionship and the benefits companionship confers.

The research on the health and quality of life benefit of companion animals in the general population is inconclusive. Companion animals can provide benefits to owners with mental health conditions [7]. Participants with dogs, cats, or both dogs and cats have reported higher general health than participants with no pets [8]. Furthermore, the American Heart Association authored a consensus statement concluding that pet ownership, particularly of dogs, is probably associated with decreased cardiovascular disease (CVD) risk. Additionally, they concluded that pet ownership, particularly of dogs, may have some causal role in reducing CVD risk [9]. However, other research, including a study of 2551 Australians aged 60–64 years, found no difference between pet owners’ and non-pet-owners’ scores of the 12-item Short Form (SF-12)’s mental composite score [10]. Some have argued that companion animals may increase feelings of wellbeing, particularly by attenuating loneliness among older adults living alone [11]. At least one study reported that pets may be of particular value to older adults recovering from major illness, and another study found particular benefit for one-year survival for patients after discharge from coronary care unit [12,13]. However, other research has found negative mental health effects associated with companion animals [14,15,16]. In a recent cross-sectional study, pet owners had higher rates of depression than participants without pets [14]. Research has also found negative associations beyond depression, including financial burden and grief over loss of the companion [15]. Results from an online, nationwide cross-sectional study indicated that pet owners were more satisfied with their lives than non-owners, but did not differ from them on a wide range of wellbeing measures, personality measures, emotion regulation, or need satisfaction [16].

Within groups who own companion animals, differences in scores of wellbeing have been observed. In a study of 42,044 people living in California, participants with dogs scored higher on all aspects of wellbeing compared to participants with cats. However, those with dogs were more likely to have higher body mass indexes (BMI) and had higher household incomes than those with cats [8]. Another study found opposite results, with dog owners having lower BMIs and less obesity than cat owners, non-owners, or both cat and dog owners [17]. Another study found that dog owners walked for a higher number of minutes than non-owners [18]. 

Because previous studies have shown both positive and negative health effects as well as no effects, we investigated what relations, if any, pet companionship has with quality of life outcomes in sexual minority prostate cancer survivors [7,8,9,10,12,13,14,15,16]. We hypothesized that participants with companion animals had different mental and physical quality of life summary scores than those without companion animals. Further, we hypothesized that participants with dogs had different physical quality of life summary scores than participants with cats or participants without pets, given that dogs need to go on regular walks, leading to more physical activity for dog owners [18].

## 2. Materials and Methods 

### 2.1. Participants

The Restore Study was an NIH-funded exploratory study to examine the incidence of clinical concerns in a cohort of gay, bisexual, and other men who have sex with men treated for prostate cancer, and specifically to estimate the sexual dysfunction effects of treatment in this cohort [19]. The Restore Study had two aims: a qualitative component to document the experience of treatment in minority men and a quantitative component which utilized a cross-sectional online quantitative survey design.

The Restore Study’s participants were recruited through an online cancer support website, Malecare.org. Malecare provides support to thousands of gay and bisexual men with prostate cancer across North America [20]. Data collection lasted for 72 days between October 2015 and January 2016. Inclusion criteria were self-identification as a gay or bisexual man, treatment for prostate cancer at some point in the past, and residence in either the United States or Canada.

After screening for initial eligibility, 434 subjects passed screening, and 417 subjects completed informed consent to participate. Before the analysis took place, 233 surveys were determined to be invalid or duplicative and removed; procedures to identify and remove fraudulent and duplicative responses were performed as previously described by Dewitt et al. [21]. After these steps, 193 surveys were determined to be valid from unique respondents. Of 193 eligible surveys, participants were excluded if they did not answer the pet ownership question (*n* = 2), had only pets that were not cats or dogs (*n* = 1), or did not identify as male (*n* = 1). The final sample comprised 189 participants.

### 2.2. Measures

The survey consisted of 15 sections with approximately 150 questions and was administered in English. Questions included measures for physical and sexual functioning and bother as well as mental wellbeing. Skip and branch patterns were used to administer only relevant questions to each participant. Demographic questions were constructed from the U.S. census. Sexual and medical characteristic questions were taken from prior research [22].

Pet ownership was determined through participant self-report. Participants were first asked, “Do you currently have any pets?” Participants who responded affirmatively were given another question asking “What kind of pets?” with options of dog, cat, or other. The pet ownership question required a response to complete the survey. The measure was added to the survey in order to determine what associations, if any, pet ownership had with survey outcomes.

Physical and mental quality of life was measured through the SF-12. The SF-12 is a generic measure of health functioning composed of two subscales, which yield summary scores ranging from 0 to 100, with 0 indicating the lowest level of health and 100 indicating the highest level of health. The SF-12 is normed at mean = 50. The two subscales are a Mental Composite Score (MCS) and a Physical Composite Score (PCS). The two-week test–retest reliability for the physical subscale was r = 0.80, and that for the mental subscale was r = 0.76 [23].

### 2.3. Analysis

Descriptive statistics were summarized using means and standard deviations for continuous variables and counts for categorical variables. ANOVA and Chi-Square testing were used to determine significance for demographic characteristics.

Participants were placed into four groups: cats only, dogs only, both (cats and dogs), and no pets (ownership). Participants who selected dog, cat, or both in addition to another pet (e.g., parrot) were assigned to the cat, dog, or both categories without regard for other pets. Only one participant who selected “other” did not have a dog or cat.

After determining that the PCS and the MCS were normally distributed, linear regression was used to compare PCS and MCS for participants with dogs, participants with cats, participants with both dogs and cats, and participants without pets, in unadjusted models. Participants without pets were used as the reference group in the first round of analysis, and participants with dogs only were used as the reference group in the second round of analysis. Adjusted analyses controlled for age, years since diagnosis, income, partnership, and race. The classification of race was measured as white or non-white because the small number of non-white participants made controlling for individual racial categories unfeasible. Partnership was determined by asking participants “Are you currently married or living with a partner?”. Data were analyzed using Stata version 14 [24].

## 3. Results

The demographic characteristics of the participants are summarized in Table A1. The average participant in this study was 63.4 years old, and pet owners were, on average, younger (*p* = 0.0002) and more likely to live with a partner (*p* = 0.04) than non-pet-owners. The average participant in this study was non-Hispanic white, male, in his 60s, and gay-identified. Participants resided in 38 states and two Canadian provinces. Ninety-eight participants (52%) had pets. Of participants with pets, 25% of the participants had only a dog, 19% percent had only a cat, and 8% had both animals. 

In Table A2, in crude analyses, differences between the means of MCS scores were significantly lower for participants with cats only (−6.10, 95% CI: −10.5, −1.76, *p* = 0.006) and for participants with dogs only (−4.46, 95% CI: −8.42, −0.50, *p* = 0.027), compared with participants without pets, who had a mean MCS of 48.5. No difference was found for participants with dogs and cats. After adjusting for age, years since diagnosis, income, partnership status, and race, the differences between the means of MCS scores remained statistically significantly lower for participants with only cats (−5.98, 95% CI: −10.50, −1.47, *p* = 0.01) and for participants with only dogs (−5.59, 95% CI: −9.66, −1.53, *p* = 0.007), compared with those without pets, who had a mean MCS of 49.6. There was no significant difference found between participants with both dogs and cats and participants without pets. 

When referenced against participants with dogs, the difference in scores for participants without pets was significant (4.46, 95% CI: 0.50, 8.41, *p* = 0.027). After adjusting for age, years since diagnosis, income, partnership status, and race, when referenced against participants with dogs, the difference in the scores for participants without pets was significant (5.59, 95% CI: 1.53, 9.66, *p* = 0.007).

Crude differences in PCS scores for participants who had pets compared with those who did not were significantly higher only for cat owners (3.56, 95% CI: 0.16, 6.96, *p* = 0.04). There was no significant difference found for participants with only dogs or participants with both dogs and cats compared to non-pet-owners, who had a mean PCS of 51.4. After adjusting for age, years since diagnosis, income, partnership status, and race, there was no significant mean difference in the PCS scores between participants with only cats, participants with only dogs, or participants with both dogs and cats, compared to participants with no pets, who had a mean PCS of 52.3. No significant difference in mean PCS scores was found for any group when referenced against participants with dogs.

## 4. Discussion

The average mental health composite score for men who have only cats and men who have only dogs was significantly lower than for those who had no pets. No difference was found between participants with cats and participants with dogs in mental health composite scores. This was consistent with the hypotheses and with companion animal research in other populations [10]. It is possible that participants who have pets obtained them in part for an expected health and quality of life benefit. Thus, participants who have higher mental health measures would not seek out pets for these benefits. Alternatively, those with pets might score lower on mental health because of added stress, for example, when sick participants must deal with caring for their pets while dealing with their illness, which may include in-patient care and financial strain. 

We hypothesized that dog owners might have better physical health, given that dogs need to go on regular walks. However, after controlling for covariates, we found no significant differences in PCS scores among dog owners compared to other participants. It could be that the exercise in dog walking is not sufficient to increase health, that older gay men may not actually walk their dogs (or have someone else do so), or that the aftermath of prostate cancer prevents men from dog-walking. Further research is necessary to provide more data about the effects of companion animals, especially in communities of gay and bisexual older adults who may have pets at a higher rate than heterosexual communities. Given the prevalence of companion animals in the United States, there is a need to gather information about the positive and negative effects on health and quality of life of having pets, because of the understudied impact companion animals might have on the hundreds of millions of people with pets, especially gay and bisexual people. Furthermore, distinct differences exist in the prevalence of dogs and cats in particular between states.

While other research has found gay men with prostate cancer have worse health-related quality of life than heterosexual men, no published research has examined whether the differences found are influenced by companion animals [25]. In examining the influence of companion animals, this study is the first of its kind to be conducted in a population of gay and bisexual survivors of prostate cancer. Additionally, the influence of companion animals on heterosexual survivors of prostate cancer has similarly not been researched. When examining the results of this study, the questions raised cannot be answered without more research into the intersection of prostate cancer and companion animals. 

Limitations of this study include the relatively small sample size. If analyses were repeated with a larger sample, different results could be observed with special acknowledgement of the increased power of statistical methods used with an increase sample size. The Restore Study was not a representative sample of gay and bisexual men, as all participants had prostate cancer among other demographic differences. The Restore Study only asked about current pet ownership and thus was not able to discern non-pet-owners from those who have never had a pet in their lifetime. Additionally, while there is little variance in the size of cats, the variance in the size of dogs is huge. The breed of dog was not captured in the Restore Study. Different breeds of dogs require different amounts of physical exercise, which could affect the physical activity of their owners. Furthermore, not all dog owners walk their dogs to required levels. Another limitation is the convenience sample from an online social support website (malecare.org), which may not be generalizable to other populations of gay and bisexual men with prostate cancer. The cross-sectional design of this study does not determine directionality of associations between pet ownership and quality of life measures. Longitudinal research could help determine aspects of directionality of the associations found, while comparative samples of sexual minorities with their heterosexual peers could directly test for differences. While it could be that pet ownership lowers quality of life, those with lower quality of life might be more likely to own pets, or some third variable affecting both pet ownership and mental health (e.g., socioeconomic status) might explain the association. 

Pending results of further research, clinicians do not have enough data to confidently recommend companion animals as a mechanism to improve the quality of life of their gay and bisexual male patients. This recommendation is echoed by The American Heart Association, which in its consensus statement about pet ownership and CVD risk, recommends that pet adoption should not be undertaken for the primary purpose of reducing CVD risk [9].

## 5. Conclusions

This study provides the first analysis of the association of companion animals with quality of life measures in older populations of gay and bisexual men who have survived prostate cancer. Companion animal ownership was associated with the mental health composite score and the physical health score. Consistent with our hypothesis, mental health composite scores were significantly different for participants who had cats only and participants who had dogs only after adjustment for covariates, when referenced against participants who did not have pets. Further research must be conducted in order to better understand the influence companion animals might have on quality of life for older populations of gay and bisexual men. 

## Appendix A

**Table A1:** Demographic Characteristics.

Title	Cats Only *n* = 36	Dogs Only *n* = 47	Both *n* = 15	No Pet Ownership *n* = 91	All Participants *n* = 189	*p* ^a^
Age in Years Mean (SD)	60.7 (6.89)	60.91 (7.21)	62.1 (9.83)	66.1 (8.09)	63.5 (8.17)	0.0002
Income ($) Mean (SD)	$81,350 ($61,285)	$82,988 ($56,890)	$78,250 ($50,201)	$86,093 ($102,004)	$84,007 ($81,700)	0.98
Years Since Diagnosis Mean (SD)	5.28 (3.68)	5.59 (4.28)	3.50 (3.55)	6.09 (5.05)	5.64 (0.34)	0.28
Partnership Count (%)						0.04
Yes	22 (61%)	30 (65%)	10 (66%)	41 (45%)	104 (55%)	
No	13 (36%)	16 (34%)	5 (33%)	49 (54%)	83 (44%)	
Race Count (%)						0.72
White	32 (89%)	42 (89%)	14 (93%)	81 (89%)	169 (89%)	
Non-White	4 (11%)	5 (11%)	1 (7%)	10 (11%)	20 (11%)	
Ethnicity Count (%)						0.88
No Hispanic Origin	35 (97%)	45 (96%)	15 (100%)	87 (97%)	182 (97%)	
Hispanic Origin	1 (3%)	2 (4%)	0 (0%)	3 (3%)	6 (3%)	
Sexual Orientation Count (%)						0.71
Gay/ Homosexual	35 (95%)	41 (81%)	13 (93%)	82 (90%)	171 (91%)	
Bisexual	2 (5%)	6 (19%)	1 (7%)	9 (10%)	18 (9%)	

^a^ ANOVA for continuous variables or Chi Square (X^2^) for significance.

**Table A2:** Short Form 12 Composite Scores for Gay and Bisexual Men (GBM) after Prostate Cancer Treatment.

Unadjusted Model *n* = 189	No Pets	Cats Only	Dogs Only	Both
SF-12: Mental Health Composite Score Mean (SD)	48.5 (10.26) ^♦^	42.34 (12.0) ^■^	44.0 (12.34) ^■^	46.05 (10.34)
SF-12: Physical Health Composite Score Mean (SD)	51.4 (8.34)	55.0 (7.19) ^■^	53.2 (8.78)	50.8 (11.4)
Adjusted Model *N* = 163	No Pets	Cats Only	Dogs Only	Both
SF-12: Mental Health Composite Score Mean (SD)	49.6 (10.26) ^♦^	43.6 (12.0) ^■^	44.0 (12.34) ^■^	48.1 (10.34)
SF-12: Physical Health Composite Score Mean (SD)	52.3 (8.34)	55.2 (7.19)	52.7 (8.78)	50.2 (11.4)

Significance *p* < 0.05 by reference group; ^■^: ref. no pets; ^♦^: ref. dogs.
